# The genotypic antibiogram: using gram-negative antimicrobial resistance genes identified via rapid blood culture identification tests to optimize treatment of Enterobacterales bloodstream infections

**DOI:** 10.1017/ash.2024.406

**Published:** 2024-10-14

**Authors:** Shawnalyn W. Sunagawa, Jeremy Tigh, Yolande A. Chan, Ashlyn Okada, Scott J. Bergman, Trevor C. Van Schooneveld, Paul D. Fey, Jonathan H. Ryder

**Affiliations:** 1 Department of Pharmaceutical and Nutrition Care, Nebraska Medicine, Omaha, NE, USA; 2 College of Pharmacy, University of Nebraska Medical Center, Omaha, NE, USA; 3 Department of Pathology, Microbiology, and Immunology, University of Nebraska Medical Center, Omaha, NE, USA; 4 Department of Internal Medicine, Division of Infectious Diseases, University of Nebraska Medical Center, Omaha, NE, USA

## Abstract

The negative predictive value of *bla*_CTX-M_ on BCID2 for ceftriaxone resistance in *E. coli* and *K. pneumoniae* group was 97% and 94%, respectively. Creation of a genotypic antibiogram led to updated local guidance for clinicians to utilize for empiric treatment of Enterobacterales bloodstream infections identified via rapid diagnostics.

Among *Escherichia coli* and *Klebsiella pneumoniae* isolates in the United States, 85.4% of extended-spectrum beta-lactamases (ESBLs) were due to *bla*_CTX-M_.^1^ With the increasing incidence of ESBL-producing isolates, rapid diagnostic tests, which include genotypic information such as the detection of *bla*_CTX-M_, have been shown to improve time to more appropriate, and even optimal, empiric therapy.^2^ This is critical since patients with ceftriaxone-resistant infections have been found to have worse outcomes.^2,3^ The BioFire BCID2 is a second-generation multiplex panel which rapidly identifies 30 different Gram-negative, Gram-positive, and yeast pathogens along with 10 antimicrobial resistance genes from blood cultures.^4^ Specifically, the *bla*_CTX-M_ resistance marker detects the presence or absence of the most common family of ESBL enzymes; however, little is known regarding the safety of de-escalation in the absence of this marker.^5^ Genotypic markers of ceftriaxone resistance appear to outperform ESBL clinical prediction rules.^6^ Thus, we created a genotypic antibiogram to use in combination with BCID2 results to assist with improving our institutional guidance for empiric antimicrobial therapy selection for patients with Enterobacterales bloodstream infections (BSI).

For this study, all positive BCID2 results with monomicrobial Enterobacterales BSIs at our academic medical center from 8/1/2021 to 11/1/2022 were retrospectively reviewed. Isolates with multiple positive resistance markers were excluded. Patient characteristics, BCID2, culture, and susceptibility results were collected. Immunocompromised patients were defined as people living with HIV with CD4 < 200, having received a solid organ or bone marrow transplant, or undergoing treatment for a hematologic and/or oncologic malignancy. Community-onset infections were defined as blood cultures collected < 48 hours from hospital admission. We performed descriptive statistics for cohort characteristics. Sensitivity, specificity, positive predictive values (PPV), and negative predictive value (NPV) of the *bla*_CTX-M_ marker was compared to ceftriaxone susceptibility. Antimicrobial susceptibility testing was performed using the MicroScan WalkAway System (Beckman Coulter) and the MicroScan Negative MIC 56 antimicrobial panels, according to the manufacturer’s instructions. ESBL and AmpC producers were identified per our institutional protocol (Supplemental Material). CLSI guidance was followed for interpreting results (M100) and creation of the antibiogram (M39) utilizing the first patient isolate per year methodology.^7^ Our institutional review board deemed this a quality improvement project exempt from review.

Over 15 months, 455 Enterobacterales bloodstream isolates were identified from 452 unique patients. Of those, 236 (52%) were male patients, 189 (41%) immunocompromised, and 342 (75%) had community-onset infections. The most common species identified were *Escherichia coli* (55%) and *Klebsiella pneumoniae* group (17%). *bla*_CTX-M_ was detected in 48 (11%) isolates from 41 (85%) *E. coli* and 6 (13%) *K. pneumoniae* group (Supplemental Table 1). *E. coli* and *K. pneumoniae* isolates that did not harbor *bla*_CTX-M_ detected were 97% and 100% susceptible to ceftriaxone, respectively. *bla*_KPC_ was detected in 1 isolate (*K. variicola*), and excluded from our analysis. Additionally, no other carbapenemase genes were detected. For *E. coli*, *bla*_CTX-M_ sensitivity and specificity for detection of ceftriaxone resistance was 85% and 100%, respectively; *bla*_CTX-M_ PPV for ceftriaxone resistance was 100%, while the NPV of absent *bla*_CTX-M_ for ceftriaxone susceptibility was 97% (Table 1). Of 7 *bla*_CTX-M_ negative *E. coli* isolates, 6 were identified as ESBL and one as an AmpC producer based on phenotypic lab protocols. For *K. pneumoniae* group, *bla*_CTX-M_ sensitivity and specificity for detection of ceftriaxone resistance was 60% and 100%, respectively; *bla*_CTX-M_ PPV for ceftriaxone resistance was 100%, while the NPV of absent *bla*_CTX-M_ for ceftriaxone susceptibility was 94% (Table 1). Among the other Enterobacterales species detected on BCID2, only one had *bla*_CTX-M_ detected despite 24% (31/129) having ceftriaxone resistance. Figure 1 describes susceptibilities to common antimicrobials, delineated on the presence or absence of BCID2 resistance markers, for *E. coli and K. pneumoniae* group since these organisms are most likely to harbor *bla*_CTX-M_, whereas other organisms are more likely to have other mechanisms of ceftriaxone resistance (eg, *Enterobacter* species and AmpC).^1^ Supplemental Figure 1 describes susceptibilities for all organisms.

**Table 1.** BCID2 organisms with *bla*_CTX-M_ detected sensitivity, specificity, positive, and negative predictive values

BCID2 Organism^a^	Total Isolates (N)	*bla*_CTX-M_ Detected by BCID2 (N)	Ceftriaxone Resistance (N)	SN (%)	SP (%)	PPV (%)	NPV (%)
*E. coli*	249	41	48	85	100	100	**97**
*K. pneumoniae* group^b^	77	6	10	60	100	100	**94**
Enterobacterales order only^c^	32	1	9	11	100	100	**74**

Note. SN, sensitivity; SP, specificity; PPV, positive predictive value; NPV, negative predictive value.

a All organisms with *bla*_CTX-M_ detected on BCID2. Those without *bla*_CTX-M_ detected are not shown.

b Includes *Klebsiella pneumoniae*, *Klebsiella quasipneumoniae*, and *Klebsiella variicola*.

c Organism cultured that was *bla*_CTX-M_ positive identified as *Providencia rettgeri*.

**Figure 1.**
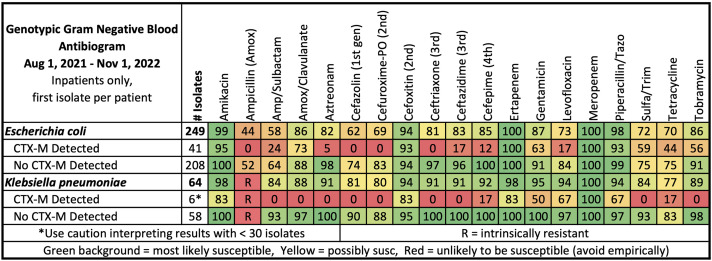
Genotypic Antibiogram.

To our knowledge, this study is the first to assess the utility of BCID2 to create a genotypic antibiogram. Previous literature focused on the Verigene Gram-negative blood culture nucleic acid test’s ability to provide empiric treatment recommendations based on the presence or absence of resistance markers.^5,8^ Our approach is novel in assessing BCID2, which contains additional resistance markers and organism targets in comparison to Verigene, providing clinicians with additional information to target antimicrobial therapy.^4^ Results from this study demonstrate similar PPV and NPVs for ceftriaxone susceptibility.^5,9^ Based on the high NPV in *E. coli* and K. *pneumoniae* group, clinicians can confidently de-escalate therapy to ceftriaxone when the *bla*_CTX-M_ resistance marker is not detected at our institution. A genotypic antibiogram (Figure 1) was distributed to clinicians simultaneously with our updated BCID2 utilization guidance document^10^ which provides empiric antibiotic recommendations based on the BCID2 results. While rapid diagnostics are useful in identifying organisms to target, clinicians often struggle to interpret these complex tests, and education and guidance on how to optimally utilize and interpret the results is continually needed.^11^ A recent study^12^ questioned the overall efficacy and diagnostic accuracy of cumulative antibiograms’ ability to predict resistance for isolates; however, genotypic blood culture antibiograms likely have improved predictive capability.

Limitations include the retrospective, single-institution design; however, this project is easily replicable and can provide other facilities with a framework for assessing their specific resistance patterns and rapid diagnostic data. Extrapolation of the results to other multiplex panels (eg, pneumonia panel) should be done cautiously. Additionally, there may be limited value of an annual genotypic antibiogram given the limited sample size and additional cost/time associated with its creation. However, genotypic antibiograms may provide insight into the monitoring of epidemiologic trends in ESBLs within an institution. Other limitations include the low number of *bla*_CTX-M_ isolates, which limits applicability to only a select number of organisms. Since the proportion of *bla*_CTX-M_ isolates was representative of the overall BCID2 results, the genotypic antibiogram still provides clinically relevant information regarding susceptibilities. Further, for our institution, absence of *bla*_CTX-M_ does not reliably predict ceftriaxone susceptibility for non-*E. coli* or non-*K. pneumoniae* isolates. Finally, antimicrobial utilization and prescriber response based on BCID2 results was not collected; however, previous literature supports that utilization of rapid diagnostics, when coupled with stewardship, reduces time to optimal antimicrobial therapy, and our stewardship program reviews BCID results daily.^13^ Further studies should be performed to explore the utilization and impact of BCID2 gram-negative resistance markers on time to optimal therapy and patient outcomes.

This study demonstrated that the majority of ceftriaxone-resistant *E. coli* and *Klebsiella* BSI harbor *bla*_CTX-M_, and carbapenemases are rare in our institution. Creating a genotypic antibiogram assisted in providing updated guidance for our clinicians on treatment of Enterobacterales bloodstream infection.

## Supporting information

Sunagawa et al. supplementary material Sunagawa et al. supplementary material

## Supplementary material

For supplementary material accompanying this paper visit http://doi.org/10.1017/ash.2024.406. click here to view supplementary material
