# Supplementary material for: The genotypic antibiogram: using gram-negative antimicrobial resistance genes identified via rapid blood culture identification tests to optimize treatment of Enterobacterales bloodstream infections
Source: Antimicrob Steward Healthc Epidemiol. 2024 Oct 14;4(1):e172. doi: 10.1017/ash.2024.406 (PMC11474757; doi:10.1017/ash.2024.406)
Supplement: Sunagawa et al. supplementary material [file S2732494X24004066sup001.docx]

**Supplemental Table 1:** Population characteristics

| **Characteristic** | **Overall (N=455)** | ***bla*_CTX-M_ Positive (N=48)** | ***bla*_CTX-M_ Negative (N=407)** |
| --- | --- | --- | --- |
| Male | 236 (52%) | 28 (57%) | 208 (51%) |
| Immunocompromised^a^ | 189 (41%) | 22 (45%) | 167 (41%) |
| Location of infection onset^b^ |  |  |  |
| Community-onset | 342 (75%) | 32 (67%) | 310 (76%) |
| Hospital-onset | 113 (25%) | 16 (33%) | 97 (24%) |
| BCID2 Result |  |  |  |
| *Escherichia coli* | 249 (55%) | 41 (85%) | 208 (51%) |
| *Klebsiella pneumoniae* group^c^ | 77 (17%) | 6 (13%) | 71 (17%) |
| Enterobacterales order only | 32 (7%) | 1 (2%) | 31 (8%) |
| *Proteus* spp. | 26 (6%) | 0 (0%) | 26 (6%) |
| *Klebsiella oxytoca* | 22 (5%) | 0 (0%) | 22 (5%) |
| Other | 49 (10%) | 0 (0%) | 49 (12%) |
| Resistance Markers |  |  |  |
| CTX-M | 48 (11%) | 48 (100%) | 0 (0%) |
| KPC | 1 (0.2%) | 0 (0%) | 1 (0.2%) |
| Final Culture Results |  |  |  |
| *Escherichia coli* | 249 (55%) | 41 (84%) | 208 (51%) |
| *Klebsiella pneumoniae* | 64 (14%) | 6 (12%) | 58 (14%) |
| *Klebsiella oxytoca* | 25 (5%) | 0 (0%) | 25 (6%) |
| *Proteus mirabilis* | 23 (5%) | 0 (0%) | 23 (6%) |
| *Enterobacter cloacae* | 21 (5%) | 0 (0%) | 21 (5%) |
| *Klebsiella variicola* | 13 (3%) | 1 (2%) | 13 (3%) |
| *Serratia marcescens* | 13 (3%) | 0 (0%) | 13 (3%) |
| Other^d^ | 47 (10%) | 0 (0%) | 47 (12%) |

^a^ Hematologic/oncologic malignancy, bone marrow transplant, solid organ transplant, human immunodeficiency virus with CD4 < 200

^b^ Community-onset: positive blood culture < 48 hours from hospital admission, hospital-onset: positive blood culture > 48 hours from hospital admission

^c^ Includes *Klebsiella pneumoniae*, *Klebsiella quasipneumoniae*, and *Klebsiella variicola*

^d^ Includes *C. freundii* (11), *K. aerogenes* (9), *Salmonella* spp. (8), *Pantoea* spp. (5), *M. morganii* (4), *P. vulgaris* (3), *Providencia rettgeri* (3), *C. koseri* (1), *C. amalonaticus* (1), *Pseudescherichia vulneris* (1), *Providencia stuartii* (1)

**Supplemental Figure 1:** Percentage of susceptible isolates


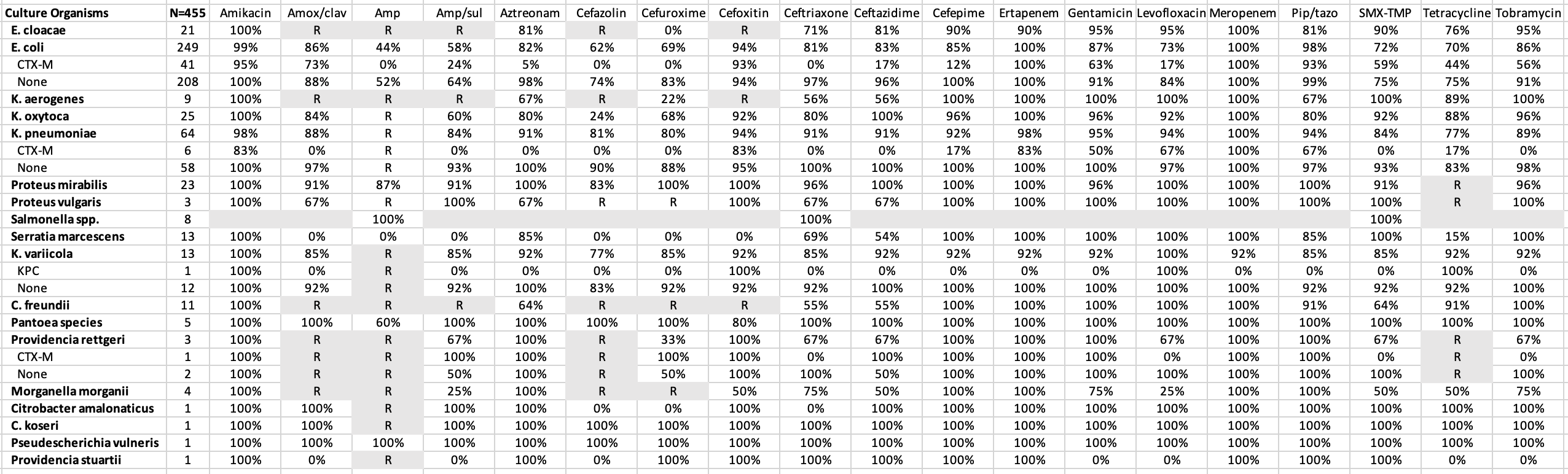


Abbreviations: Amox/clav = amoxicillin/clavulanate, amp/sul = ampicillin/sulbactam, pip/tazo = piperacillin/tazobactam, TMP-SMX = trimethoprim/sulfamethoxazole, R = intrinsically resistant, - = susceptibilities not routinely performed

**UNMC Phenotypic Lab Protocol**

For *E. coli*, *K. pneumoniae*, *K. oxytoca*, and *P. mirabilis* isolates, the presence of an ESBL was determined by comparing the MIC values of ceftazidime to ceftazidime/clavulanate (4 μg/mL) or cefotaxime to cefotaxime/clavulanate (4 μg/mL). An ESBL was considered present if the MIC of a single antimicrobial was ≥ 3 doubling dilutions higher than the MIC of the same antimicrobial when combined with clavulanate. Only one comparison was needed to meet the criteria for a positive ESBL. For isolates that were deemed possible ESBL-producers but could not be confirmed due to the limited dilutions of the panel (i.e. the upper limit of the range was not high enough), an ESBL confirmation test was performed according to Clinical and Laboratory Standards Institute (CLSI) guidance (M100). Briefly, a disk diffusion method was utilized in which a standard inoculum of the isolate (0.5-McFarland) was prepared and plated as a lawn on Mueller Hinton agar. Disks containing ceftazidime (30 μg), ceftazidime/clavulanate (30/10 μg), cefotaxime (30 μg), and cefotaxime/clavulanate (30/10 μg) were placed on the agar at a defined distance. Following incubation at 35°C for 16–18 h, the zones of inhibition were measured. A positive ESBL result was defined as an increase in zone diameter of 5 mm or more for ceftazidime/clavulanate or cefotaxime/clavulanate compared to the zone diameter for the single antimicrobial agent. For ESBL-negative isolates of *E. coli*, *K. pneumoniae*, *K. oxytoca*, and *P. mirabilis*, the presence of AmpC was defined by the following criteria: (1) intermediate or resistant to cefotaxime, ceftazidime, or ceftriaxone; (2) intermediate or resistant to cefoxitin; and (3) susceptible to cefepime.
